# The Insane Asylums of Texas—Need of Reform

**Published:** 1900-07

**Authors:** 


### The Insane Asylums of Texas—Need of Reform.

Letter from Superintendent Worsham of the Austin asylum to the Governor:

STATE LUNATIC ASYLUM,
AUSTIN, TEXAS, June 14, 1900.

*Governor J. D. Sayers, Austin, Texas:*

DEAR SIR: Knowing your desire to have the State properly provide for all of the insane within her borders, your interest in having laws enacted that might better their condition, and at the same time more economically meet these ends, is my excuse for addressing you this communication, and in it call your attention to changes in the present law governing the admission of patients into the insane asylum and their management that in my judgment would be beneficial.

The statutory laws under which our asylums are now being conducted were passed many years ago and perhaps, with a few exceptions, remain unchanged, while the condition and the opinions as to the proper management of such institutions have materially changed.

When asylums were first organized the opinion then prevailed that insanity was not a disease, but a curse placed upon the individual by divine power as a punishment for violating some laws of God. The object of the asylum was not the care and treatment, but for the confinement of the lunatics and the protection of society and the public generally from this class. And upon that theory all who were admitted as inmates had to be first carried before the proper court, tried by a jury, convicted and sentenced to the asylum.

It has long since been demonstrated and accepted that insanity is a disease, and that all persons thus affected require hospital care and treatment. So that today the former madhouse or lunatic asylum is transformed into a hospital for the care and treatment of people suffering from mental disease, yet the jury trial of the ancients is in full force in Texas. All the States in the United States with only a few exceptions have abandoned this procedure of commitment and admit their insane into the hospital in a more humane way. To take a lady or gentleman who is suffering from an acute attack of insanity into open court, frequently handcuffed, or tied, in the custody of the sheriff, force them to be an object of curiosity and go through the form of trial that criminals do, is a relic of barbarism practiced upon these unfortunates in the darker ages that should be remedied. The cost of the jury trial in causes of insanity amounts to considerable, to say nothing of the humiliation to the relatives and the absolute harm that results to the unfortunate patient. They get the impression (a large number of them) that they have been unjustly accused of some crime, tried and convicted.

This idea is a constant worry to them, and in cases that recover, the knowledge that publicity has been given to their misfortune by the court trial is a source of annoyance and humiliation.

A plan that would be free from the above objections, more economical and freer from the mistakes that are frequently made under the present system by convicting persons who are not insane, or not proper subjects for admission as patients into the asylum, would be to have the law, first, more clearly define the character of persons who are entitled to care and treatment in the asylum, and in that way relieve the State of the burden of caring for patients who do not require asylum care. Under the present system there is a growing tendency on the part of relatives and friends to place in the asylum all classes of defective persons in order to relieve themselves of their care. Many old and decrepit persons who are helpless but suffering from no mental trouble except mental enfeeblement (a natural result of their advanced age), are technically declared insane and placed in the asylum as dangerous lunatics. This is an unjust burden to the State and an injustice to the person so confined, as all they need is slight care and attention, which could easily be given by their relatives and friends at home.

Then when the person is to be committed to the asylum require the guardian, one or more relatives, friends or acquaintances, as the individual case may require, to go before the county judge, make formal application for admission of the patient to the asylum, and have two reputable physicians, preferably the family physician and county physician, to make sworn certificates that the person to be admitted is a proper one for asylum care and treatment, and to furnish as complete clinical and family history of the case as possible, to be kept as a record of the case at the institution.

The argument might be brought against this form of commitment that it would be too easy to get a patient into the asylum, and as a result patients who are not insane might be placed there by relatives and friends. But this can easily be set aside when we consider that it is not only the privilege, but the duty, of the superintendent to discharge any person confined in the asylum, when, in his opinion, the person is not insane or not a proper subject for restraint.

In conveying patients to the asylum, it should be done by relatives or friends when at all practicable, and no female patient should be carried any distance to the asylum except when accompanied by at least one female attendant. Under the present system, patients are carried to the asylum in the same way that convicts are to the penitentiary; namely, in the custody of the sheriff or some other officer, frequently handcuffed or otherwise restrained.

A feature concerning the management of all our eleemosynary institutions that in my judgment cripples the efficient management of them, pertains to Article 94, Revised Statutes, in which, among other things, the board of managers is given the power to fix the salaries of all officers and employes of their respective institutions. This is as it should be, but the legislature, in making appropriations, have for a number of years past rendered this inoperative by reason of the fact that they stipulate the salary to be paid each person, from the lowest to the highest. The injustice of this procedure can readily be understood when we understand the fact that attendants and nurses require special training and experience before their services are worth anything to the institution. We are forced now to pay as much to the inexperienced beginners the first month of their service as they receive after months or years of training and experience. The efficient, good, bad and indifferent are all paid the same from the start to the finish of their service.

Under such a rule, there is no incentive to improve or advance in their work, and as a result the management is constantly forced to make changes among the employes, which is a serious detriment to the proper management of the institution and the welfare of the patients.

The amount of salaries of employes should be made in bulk and the board of managers and superintendent allowed to exercise the right to grade them according to merit and length of service. Start the beginners on a very small salary and advance them gradually as demanded by merit. This plan would not cost in the aggregate as much, or certainly no more, than the present system, and the decided improvement to the service would be beyond question.

Another feature that has been the custom in making the appropriations for the various institutions that works an unnecessary hardship upon their proper management is making the appropriation under different headings, or items of expense, such as groceries, water, fuel and lights, dry goods, drugs, repairs, etc., fixing the amount allowed for each special heading. Very frequently it is found that one item of appropriation is not sufficient to meet the needs in that particular line, while another is in excess of what is needed, and as a result one department suffers, and at the next session of the legislature a larger appropriation is necessary by reason of this deficiency, and the fact that the emergency was not promptly met at the proper time. This is notably true in the item of repairs. No man can anticipate the needed repairs for an institution of this character for two years, and I do not think any one will attempt to make the argument that it is not in the interest of economy to keep public buildings in good repair. When repairs are neglected, it costs far more in the end than it would to keep them up when occasion demanded. The amount of drugs required depends upon the health of the patients. During the past few months it seemed absolutely necessary to vaccinate the entire population of this institution, but under the present arrangement of the appropriation it was impossible to do so on account of the limited appropriation for drugs.

By making a careful estimate, based upon the per capita cost of previous years, and making such an appropriation in bulk for the maintenance of each institution, the aggregate, in my opinion, would be less and there would be no more show of possibilities of mismanagement than under the present system. Every department could be carried on equally, and it would be a decided stimulus to better management. Nearly all of the States make the appropriation for the institutions in bulk, and the cost of maintenance is no more than it is in our State.

With reference to this particular institution, I desire to call your especial attention to the fact that the farm is too small to meet our urgent needs. We should have at least 500 acres more land so that we could utilize all of the patients' labor possible, which would be a great benefit to them, and an immense saving to the State in supporting the institution. By investigating the methods of the other Southern institutions, I find that many of them have adopted extensive farming and gardening industries, and the plan works so admirably that others are preparing to adopt the same principle. The plan universally accepted as being the best, is to construct inexpensive cottages on the farm separate and distinct from the main building, and there colonize all of the male working patients. Alabama has had this plan in operation for several years and has fully demonstrated the benefits of such assistance. North Carolina and Georgia will soon be ready to inaugurate the same plan. At the Alabama institution cottages have been erected to accomodate 100 patients, with kitchen, barn, dairy and everything that goes with the equipment of a first-class farm. They have about two thousand acres of land, and the amount of produce they raise is simply astonishing to those who have never thought about the possibilities in this line. They raise all feed necessary to maintain dairies, large enough for the entire institution, which has a population of over 1,500. They have their own mills for grinding corn to supply all of the cornmeal used by the institution. They buy their beef on foot, fatten the same with feed raised on the farm and slaughter their own meat. While Texas, the greatest stock raising and farming country on earth continues to buy a large amount of feed for dairy cattle; the dairy is not large enough to supply the demand of the institution. The above is only a list of the larger items made possible by a large and well managed farm, not to mention the possibilities of raising large quantities of vegetables and fruit that could be used in season and canned and otherwise stored for future use.

By comparison, I will mention the per capita cost of the Alabama institution was $104, while at this institution it was $135.92, and this is the lowest per capita this asylum has ever reached within its history.

It is fair to state, however, that we could never expect to reach so low a per capita as Alabama, as they own their own coal mines near the institution and mine their own fuel with patients' labor. The argument might be brought up that the price of land is so high near this institution that it would not be a good investment for the State, but I am sure that when any practical business man understands the fact, that the cost of cultivating the land is almost nothing to the State, it will be regarded as not a bad investment, even though the price of land be high. Land that, we may say for instance, cost $40 per acre, it would be reasonable to expect that from 25 to 35 bushels of corn to the acre would not be an unusual crop. This amount of corn at the present market price of 50c would exceed 30 to 40 per cent. on the investment, to say nothing of the benefit derived to the patients by employment. By colonizing the males who can work separates them from the noise and confinement of the wards where they are forced to be constantly associated with the violent and noisy class, and that alone is an incentive in getting them to engage in work, this plan furnishes healthful environment, and the employment relieves them of surplus energy that is liable to be expended by violent outbreaks in many and is the best remedy for the melancholy and depresesd condition of others.

The State could easily procure land within a reasonable distance to the main building, and a distance of a mile or two is no objection. Construct inexpensive farm houses and organize a colony with at least 100 working patients, fifty whites and fifty colored.

Your attention is called to this fact that for a number of years no provision has been made to increase the room for colored patients at this institution. For several years past it has been impossible to accommodate but few of this class. It will be absolutely necessary to provide more room for them at the next session of the legislature, and the above plan is by far the most economical, and will result in great saving in cost of maintenance.

It is my candid opinion that in the future it will be found necessary to colonize all of the chronic insane who are able to work, and then make them as near self-sustaining as possible.

That there are great possibilities in the way of lessening the cost of maintenance of these institutions upon the lines indicated, I have not the least doubt, and in view of the fact that the care and support of the dependent insane is a growing burden upon the people of every State, I think that Texas should have the benefit of every advancement that might tend to lessen this burden, and at the same time, render the lives of these, the most unfortunate of all classes, more bearable and useful.

Most respectfully,

B. M. Worsham,

Superintendent State Lunatic Asylum.

